# A retrospective study of *Taenia* spp. in Cuban patients: what does molecular analysis tell us?

**DOI:** 10.1016/j.fawpar.2021.e00136

**Published:** 2021-10-15

**Authors:** Luis Enrique Jerez Puebla, Fidel A. Núñez Fernández, Jorge Fraga Nodarse, Raul Cordovi Prado, Iraís Atencio Millán, Iredys Cruz Rodríguez, Rigoberto Fimia Duarte, Marina del Carmen Sánchez Romero, Sahily de la Caridad Ortega Medina, Ubaldo del Risco, Lissette Pérez Santos, Lucy J. Robertson

**Affiliations:** a Departamento de Parasitología, Instituto "Pedro Kourí", Autopista Novia del Mediodía Km 6½ e/Autopista Nacional y Carretera Central, La Habana, Cuba; b Latin-American School of Medical Science, La Habana, Cuba; c Faculty of Health Technology and Nursing, University of Medical Sciences of Villa Clara, Cuba; d Hygiene Provincial Centre of Microbiology and Epidemiology, Guantánamo Province, Cuba; e Hygiene Provincial Centre of Microbiology and Epidemiology, Cienfuegos Province, Cuba; f Hygiene Provincial Centre of Microbiology and Epidemiology, Camagüey Province, Cuba; g Department of Virology, Tropical Medicine Institute "Pedro Kourí", Autopista Novia del Mediodía Km 6½ e/Autopista Nacional y Carretera Central, La Habana, Cuba; h Faculty of Veterinary Medicine, Norwegian University of Life Sciences, Ås, Norway

**Keywords:** Cuba, Human infection, Diagnosis, Epidemiology, Molecular confirmation, *Taenia*, Taeniosis

## Abstract

Taeniosis is a neglected disease, particularly in developing countries, and is caused by infection with the adult tapeworm of either *Taenia solium, Taenia saginata*, and *Taenia asiatica*. Of these, *T. solium* is of primary concern due to the potential for cysticercosis should *T. solium* eggs be ingested. In Cuba, all cases of taeniosis are assumed to be caused by *T. saginata*, although some cases of cysticercosis have been documented. It is therefore important to gain further insights regarding the species causing taeniosis in Cuba, especially as diagnostic records indicate an increasing incidence, with the highest number of cases reported in 2020. In this study, we analysed 37 *Taenia*-positive faecal samples (or proglottids isolated from faecal samples) from the period 2001 until 2020 from all regions of the country. Genomic DNA was extracted from the samples, which had been stored in 10% formalin, using the QIAamp Tissue Kit. Species identification was carried out by duplex real-time PCR targeting the mitochondrial DNA. All cases were found to be *T. saginata,* and sequence analysis of three isolates confirmed the identification of this species. Our data do not provide any evidence that *T. solium* currently occurs in Cuba. However, given the relatively low number of samples analysed here, that the parasite may be imported with visitors or travellers who have been in endemic countries, and that taeniosis has relatively mild symptoms and thus infected patients may not seek medical attention, we recommend species determination for all taeniosis cases reported in Cuba.

## Highlights

• The incidence of taeniosis cases in Cuba is rising, with 2020 being a peak year
• Although assumed to be *Taenia saginata*, this has not been confirmed
• We investigated 37 samples from 2001 until 2020 that had been stored in formalin
• Duplex real-time PCR indicated *T. saginata*; 3 cases were confirmed by sequencing

## 1 Introduction

Taeniosis, human infection by the adult worms of *Taenia* spp., is an important concern worldwide, especially in developing countries where sanitation may be sub-optimal, grazing livestock may have access to human faeces, and there may be other risk factors for infection (Hotez et al., 2008). This disease is caused by the zoonotic tapeworms *Taenia solium*, *Taenia saginata*, and *Taenia asiatica*, all of which have humans as their definitive host, and cattle (*T. saginata*) or swine (*T. solium, T. asiatica*) as their intermediate hosts. Thus, humans harbour the adult worm in their intestines, and eggs are released into the environment by shedding of proglottids in the faeces. Upon ingestion of the tapeworm eggs by the relevant intermediate hosts, the oncospheres hatch in the intestine, and migrate through the intestinal wall, developing into metacestode larvae (cysticerci) in the striated muscles and other organs. The adult worms develop in the intestines of people who ingest these cysticerci in raw or undercooked meat.

Of the three species, *T. solium* is of greatest public health concern due to the potential for cysticercosis, in which people, as well as intermediate hosts, may be infected by eggs shed by the mature worm, and which can develop into the metacestode cysticerci in different organs. *T. solium* infections, including cysticercosis, is rarely reported from those countries where pork is not consumed (Nakao et al., 2002), whereas *T. saginata* has been reported from all continents (except Antarctica). *T. asiatica* cases are predominantly reported from Asia (Flisser, 2006; Eom et al., 2009).

Intestinal taeniosis is frequently asymptomatic and, where symptoms are present, these are usually mild and non-specific. Abdominal discomfort, weight loss, diarrhoea, constipation, and abdominal pain may arise when the tapeworms become fully developed in the intestine, 6–8 weeks after ingestion of the meat containing cysticerci (Mendlovic et al., 2021). Symptoms may continue until the tapeworm dies following treatment, otherwise it may live for years (Hotez et al., 2008). Occasionally appendicitis or cholangitis can result from migrating proglottids (Hotez et al., 2008).

Cysticercosis, resulting from ingestion of *T. solium* eggs, and development of the metacestode cysticerci may cause much more severe symptoms, that vary depending upon the locations and numbers of cysticerci in the host. Potential locations include skeletal and heart muscle, skin, subcutaneous tissues, the lungs, liver, and other tissues, including the oral mucosa; in most locations the symptoms may be diffuse. However, cysticerci may also localise in the CNS, including brain tissue, meninges, and spine, where they may cause neurocysticercosis (NCC). NCC can be associated with serious neurological and epileptic manifestations, and may result in death, with symptoms arising when the parasites start degenerating and activate inflammatory immune responses (Mendlovic et al., 2021). Due to the life cycle of *T. solium*, cysticercosis can only occur where taeniosis occurs, as the infected human will be the source of eggs causing cysticercosis; the presence of a *T. solium*-carrier in the household is the main risk factor for cysticercosis (García-García et al., 1999). Thus, it is important to distinguish between taeniosis caused by *T. solium* or caused by *T. saginata* or *T. asiatica*.

Diagnosis of taeniosis is mostly initially made by observation of proglottids shed in stool samples, and may be aided by microscopic identification of eggs and/or proglottids. As eggs of *Taenia* spp. are morphologically identical, speciation is not possible if solely based on microscopic examination of eggs (Mwape and Gabriël, 2014). Species can be indicated by inspection of gravid proglottids and counting of uterine branches, which can be improved by injecting black ink into the genital atrium to improve visualisation; however, overlap between the numbers of uterine branches from each species has been reported and thus there may be uncertainty (Mwape and Gabriël, 2014). Species can also be determined by examination of the tapeworm scolex, as *T. saginata* does not have rostellar hooks, but these occur on the rostella of both *T. solium* and *T. asiatica*. Molecular tools that enable *Taenia* species-specific detection have been developed using primers targeting nuclear ribosomal (18S rDNA) and mitochondrial DNA (e.g., cytochrome *c* oxidase subunit 1 (cox1)) for the identification of *Taenia* tapeworms and differentiation by sequencing, PCR-restriction fragment length polymorphisms (PCR-RFLP) and multiplex PCR (Ale et al., 2014).

In Cuba, reporting of taeniosis is obligatory and, according to the Zoonosis Group of the Cuban Ministry of Public Health, taeniosis is one of the prominent emerging zoonoses in recent years (MINSAP, 2018). In national surveys of intestinal parasitic infections conducted in 1984 and 2009, *Taenia* spp. had a prevalence of 0.04% and 0.1%, respectively (Rojas et al., 2012), but speciation was not attempted. Although a handful of cases of cysticercosis have been reported (as summarised by Kanobana et al., 2013), it is not always clear where the person was infected. It has been speculated the apparently autochthonous cases were likely to be imported; three of five autochthonous cases of cysticercosis were diagnosed in Baracoa, which is remotely located on the Eastern tip of Cuba, but Haitians attempting to reach USA sometimes arrive there unintentionally (Kanobana et al., 2013). In general, it is assumed that all cases of autochthonous taeniosis in Cuba are caused by *T. saginata*, but confirmatory data are lacking. Indeed, throughout the Caribbean and the Americas, human *Taenia* infections are very rarely diagnosed to the species level (Braae et al., 2018).

In order to fill this gap in our knowledge, we undertook a retrospective molecular survey on *Taenia* spp*.-*positive samples (either faeces containing eggs or proglottids or isolated proglottids) that had been preserved in 10% formalin at the National Reference Laboratory of Intestinal Parasitic Infection, ‘Pedro Kouri’, from Institute of Tropical Medicine (IPK) to determine species of *Taenia* tapeworms.

## 2 Material and methods

### 2.1 Samples included in the study

This retrospective study investigated all *Taenia*-positive samples (eggs and/or proglottids) preserved at the National Reference Laboratory of Intestinal Parasitic Infection, IPK between 2001 and 2020. In total, 37 individual samples were used. Epidemiological data, such as year of diagnosis, patient sex and age, and province of origin, were obtained from the laboratory records.

### 2.2 DNA extraction

DNA was isolated from the samples as previously described by Verweij et al. (2009) for isolating DNA from faecal samples thought to contain *Strongyloides* larvae. Briefly, approximately 200 μg faeces or proglottid sample were first washed several times in distilled water to remove the formalin, then suspended in 200 μl of PBS containing 2% polyvinylpolypyrrolidone (PVPP; Sigma, Steinheim, Germany) and heated for 10 min at 100 °C. This was followed by incubation with sodium-dodecyl sulphate-proteinase K (2 h at 55 °C), before DNA was isolated using the QIAamp Tissue Kit spin columns (QIAgen, Hilden, Germany). DNA was eluted into 200 μl molecular-grade water and stored at −20 °C before use.

### 2.3 Duplex real-time PCR

Amplification reactions were performed in a final volume of 25 μl with PCR buffer (Hotstar Taqmaster mix; QIAgen), 5 mM MgCl_2_, 2.5 μg bovine serum albumin (Roche Diagnostics Nederland B.V., Almere, Netherlands), according to the protocol of Praet et al. (2013), using *T. solium*-specific primers (TsolITS_145F and TsolITS_230R) that should amplify an 86-bp fragment of the ITS1 sequence and *T. saginata*-specific PCR primers (Tsag_ITS_F529, reverse primer Tsag_ITS_R607) that should amplify a 79-bp fragment of the ITS1 sequence. Primers were obtained from Eurogentec (Seraing, Belgium). The Tsol_ITS_169Tq_FAM double-labelled probe (Biolegio, Nijmegen, Netherlands) was used to detect *T. solium*-specific amplification. and the *T. saginata*-specific double-labelled probe Tsag_ITS_581Tq_Quasar705 (Biolegio). Sequences of primers and probes are included in Table 1. Amplification consisted of 15 min at 95 °C followed by 50 cycles of 15 s at 95 °C, 30 s at 60 °C. Negative and positive controls for both *T. saginata* and *T. solium* were included in each amplification run (Praet et al., 2013).

**Table 1:** Overview of primer and probe sequences used in the PCR analyses.

Target	Forward primer	Reverse Primer	Probe
Duplex real-time PCR (Praet et al., 2013)			
*Taenia saginata*	5’-GCGTCGTCTTT GCGTTACAC-3′	5’-TGACACAACCG CGCTCTG-3′	Quasar705–5’-CCACAGCACC AGCGACAGCAGCAA-3’-BHQ2
*Taenia solium*	5’-ATGGATCAATC TGGGTGGAGTT-3’	5’-ATCGCAGGGTA AGAAAAGAAGGT-3’	FAM-5’-TGGTACTCTGCT GTGGCGGCGG-3’-BHQ 1
Conventional PCR (Nunes et al., 2005)			
*Taenia saginata* and *Taenia solium*	5’-TGGGTTAGAT GTTAAGACGGC-3′	5’ AAAACACCCA CTAAAAGCAGA-3′	Not relevant

### 2.4 Conventional PCR for phylogenetic analysis

For 10 isolates, conventional PCR using Cox-1/F and Cox-1/R primers targeting a mitochondrial DNA (mtDNA) fragment of 521-bp from *Taenia saginata* and *T. solium* was carried out as described by Nunes et al. (2005). The PCR protocol consisted of initial denaturation of 95 °C for 5 min, followed by 35 cycles of denaturation (60 s at 95 °C), annealing (90 s at 65 °C), and extension (90 s at 72 °C), plus one cycle of 5 min at 72 °C (Nunes et al., 2005). The final volume for the PCR reaction was 25 μl. Primer sequences are included in Table 1.

### 2.5 Sequencing of PCR products and phylogenetic analysis

The DNA products amplified by the conventional PCR (Section 2.4) were purified using QIAquick® PCR Purification kit (QIAGEN Ltd.) and sequenced in both directions with the Beckman Coulter Genomics sequencing system (Essex, United Kingdom). Sequences were obtained from three samples and aligned using BioEdit v7.0.1 package, then compared with sequences in GenBank (http://blast.ncbi.nlm.nih.gov/Blast.cgi). Distance-based analyses were conducted using Kimura2-parameter distance estimates including alignments obtained from Clustal W using the Molecular Evolutionary Genetics Analysis version 6.06 (MEGA 6) (Tamura et al., 2013). Bootstrap proportions were calculated by the analysis of 3000 replicates of the phylogenetic tree.

The generated sequences were deposited in the GenBank database under accession numbers from MZ457000 to MZ457002. Reference sequences used for *Taenia* spp. were *Taenia saginata* (GenBank no. AB465244), *Taenia solium* (AB066487), and *Taenia asiatica* (AB597284); *Echinococcus granulosus* (KU601616) was used as an out group.

## 3 Results

### 3.1 Overview and epidemiological data

**Fig. 1:**
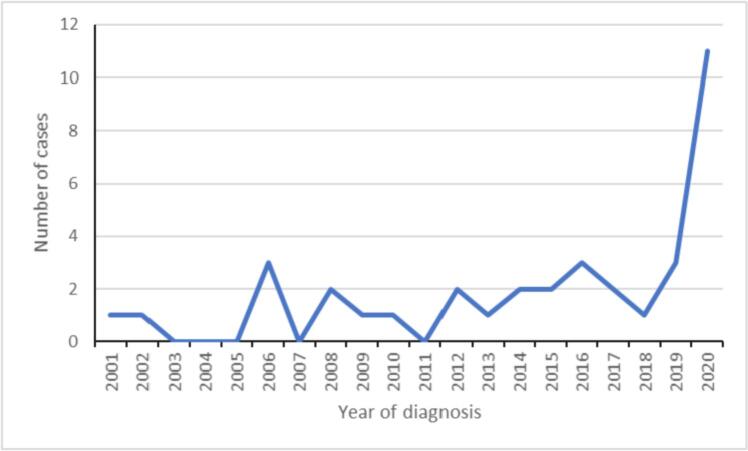
Diagnosis of taeniosis over time at the National Reference Laboratory of Intestinal Parasitic Infection, ‘Pedro Kourí’ Institute of Tropical Medicine, La Habana, Cuba.

Epidemiological data from *Taenia*-infected patients in this cohort revealed that males (25, 67.6%) were more frequently found to be infected than females (12, 32.4%). The median age was 36 years (ranging from 17 to 56 years) (Table 2). Western, central, and eastern regions of the country were represented among the samples investigated. Our data suggest that, in general, diagnosis of taeniosis at our laboratory has remained constant over the last two decades, albeit with a slightly increasing trend, and a major spike upwards in 2020 (see Fig. 1).

**Table 2:** Epidemiological data of patients (2001−2020) with laboratory-confirmed taenioisis following coprological examination at the National Reference Laboratory of Intestinal Parasitic Infection, IPK, Havana, Cuba.

Case codes	Year of diagnosis	Sex	Age (years)	Province	Results from stool examination	Conventional PCR and sequencing results
T-1	2001	F	21	Havana	Proglottids of *Taenia* sp.	Weak band – not sequenced
T-2	2002	M	47	Havana	Eggs and proglottids of *Taenia* sp.	Weak band – not sequenced
T-3	2004	F	31	Havana	Proglottids of *Taenia* sp.	Weak band – not sequenced
T-4	2006	M	35	Havana	Proglottids of *Taenia* sp.	Weak band – not sequenced
T-5	2006	F	27	Havana	Proglottids of *Taenia* sp.	Weak band – not sequenced
T-6	2006	M	51	Havana	Eggs and proglottids of *Taenia* sp.	Weak band – not sequenced
T-7	2008	M	33	Guantánamo	Proglottids of *Taenia* sp.	Weak band – not sequenced
T-8	2008	M	29	Pinar del Río	Proglottids of *Taenia* sp.	Weak band – not sequenced
T-9	2009	M	17	Havana	Eggs of *Taenia* sp.	Weak band – not sequenced
T-10	2010	F	23	Havana	Proglottids of *Taenia* sp.	Weak band – not sequenced
T-11	2012	F	41	Artemisa	Proglottids of *Taenia* sp.	Successfully sequenced
T-12	2012	M	49	Camagüey	Proglottids of *Taenia* sp.	Weak band – not sequenced
T-13	2013	M	38	Camagüey	Proglottids of *Taenia* sp.	Weak band – not sequenced
T-14	2014	M	25	Camagüey	Proglottids of *Taenia* sp.	Weak band – not sequenced
T-15	2014	F	56	Havana	Proglottids of *Taenia* sp.	Weak band – not sequenced
T-16	2015	F	43	Havana	Proglottids of *Taenia* sp.	Poor sequencing result
T-17	2015	F	34	Camagüey	Proglottids of *Taenia* sp.	Weak band – not sequenced
T-18	2016	M	27	Cienfuegos	Proglottids of *Taenia* sp.	Weak band – not sequenced
T-19	2016	M	36	Pinar del Río	Eggs of *Taenia* sp.	Poor sequencing result
T-20	2016	M	41	Pinar del Río	Proglottids of *Taenia* sp.	Weak band – not sequenced
T-21	2017	M	30	Artemisa	Proglottids of *Taenia* sp.	Weak band – not sequenced
T-22	2017	M	48	Pinar del Río	Proglottids of *Taenia* sp.	Weak band – not sequenced
T-23	2018	M	35	Pinar del Río	Proglottids of *Taenia* sp.	Weak band – not sequenced
T-24	2019	M	46	Havana	Proglottids of *Taenia* sp.	Weak band – not sequenced
T-25	2019	M	28	Pinar del Río	Proglottids of *Taenia* sp..	Weak band – not sequenced
T-26	2019	M	39	Camagüey	Proglottids of *Taenia* sp.	Poor sequencing result
T-27	2020	M	28	Camagüey	Proglottids of *Taenia* sp.	Poor sequencing result
T-28	2020	M	26	Guantánamo	Proglottids of *Taenia* sp.	Weak band – not sequenced
T-29	2020	M	35	Cienfuegos	Proglottids of *Taenia* sp.	Weak band – not sequenced
T-30	2020	M	39	Cienfuegos	Proglottids of *Taenia* sp.	Weak band – not sequenced
T-31	2020	M	33	Cienfuegos	Proglottids of *Taenia* sp.	Weak band – not sequenced
T-32	2020	F	28	Cienfuegos	Proglottids of *Taenia* sp..	Poor sequencing result
T-33	2020	F	43	Cienfuegos	Proglottids of *Taenia* sp.	Poor sequencing result
T-34	2020	M	50	Camaguey	Proglottids of *Taenia* sp.	Poor sequencing result
T-35	2020	M	51	Camaguey	Proglottids of *Taenia* sp.	Successfully sequenced
T-36	2020	F	43	Camaguey	Proglottids of *Taenia* sp.	Successfully sequenced
T-37	2020	F	47	Camaguey	Proglottids of *Taenia* sp.	Poor sequencing result

### 3.2 Confirmation of species by real-time PCR

**Fig. 2:**
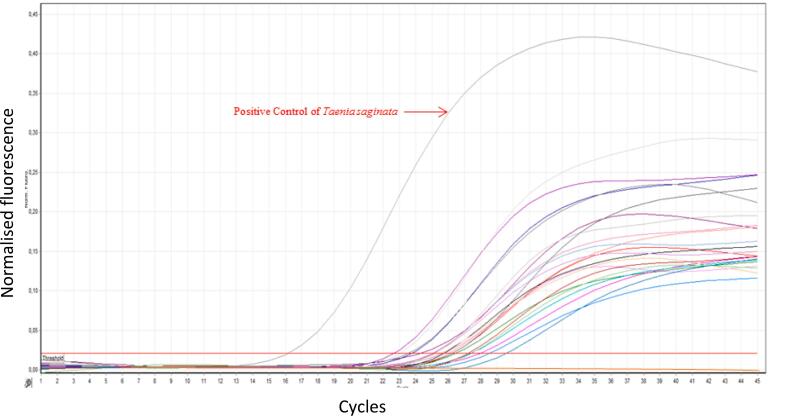
Detection of *Taenia saginata* in stool samples by real-time PCR following the protocol of Praet et al., 2013. The figure shows the positive control for *T. saginata* (indicated) and representative positive samples.

Molecular investigation by real-time PCR showed that all 37 samples were *T. saginata* (Fig. 2). No amplification of *Taenia solium* was observed in any of the samples.

### 3.3 Phylogenetic analysis of 3 samples with DNA amplification by conventional PCR and sequencing

**Fig. 3:**
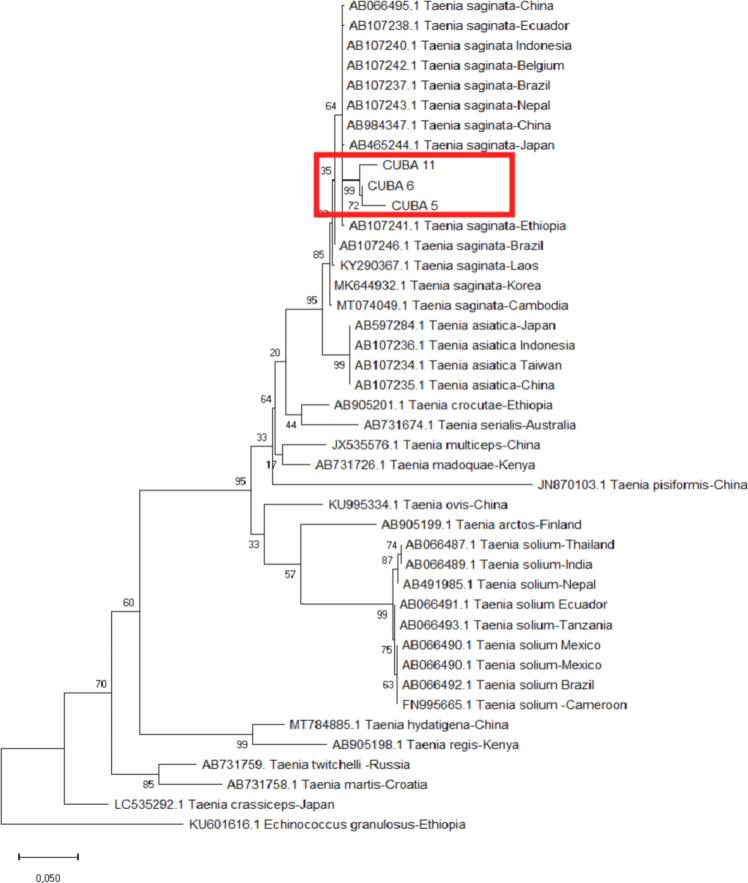
A phylogenetic tree based on *cox*1 gene sequences of PCR products using maximum likelihood analysis based on Tamura Nei Model+ Gamma Distributed (G) genetic distance model. Bootstrap analysis was performed with 3000 replications. The mitochondrial cox1 gene sequence of *Echinococcus granulosus* (KU601616) was used as an outgroup.

Following conventional PCR, 10 samples were considered to have strong enough bands for amplicon purification and sequencing. Of the amplification products submitted from 10 samples, sequences were obtained from three samples that were suitable for phylogenetic analysis. Our three sequences (MZ457000-MZ457002) clustered with the *T. saginata* cox1 genes. BLAST analysis using these sequences showed 98.02–98.24% homology with *T. saginata* isolates from Brazil (GenBank no. AB106237), Ecuador (AB107238), China (AB984347), Japan (AB465244), Ethiopia (AB107241,) and Belgium (AB107242); Fig. 3. No significant genetic differentiation among sequences from different countries was observed.

## 4 Discussion

Taeniosis due to *T. saginata* infection is an important public health concern globally, and has been reported in more than 77-million people worldwide, particularly in areas of Central and East African Africa, Asia, and highly endemic areas in the Mediterranean Region and the New World (Hiko and Seifu, 2018).

In Cuba, reporting of taeniosis is obligatory and, according to data of the Zoonoses group of the Ministry of Health, there has been an increase in the number of notified cases around the country in recent years (MINSAP, 2018). Our data supports this, with, in particular a spike in the number of cases in 2020. As some of these cases seem to have occurred in clusters from specific locations, specifically Camagüey and Cienfuegos, they may represent outbreak events where a group of individuals were infected at the same meal at which undercooked beef from an infected cow was served. Also, we cannot exclude improvements in the system for notification from regional laboratories influencing the MINSAP data or, possibly, that testing has increased.

Our data also suggest that taeniosis may occur more commonly among males than females, with more than twice as many samples from males than females in our laboratory at IPK. An association between males and *T. saginata* taeniosis has been reported from other locations (e.g., India; Lateef et al., 2020, Thailand; Anantaphruti, 2013). However, in these instances the sex distribution was noted in survey-type studies, and we cannot rule out that in our location males are more likely to seek diagnosis for this infection than females.

Until now it has been generally believed that *T. saginata* is the only species that circulates in Cuba, and porcine cysticercosis has never been detected at abattoir screenings (Dr. Mendez and Dr. Rafmari, Instituto de Medicina Veterinaria, personal communication). Our data support the hypothesis that while *T. saginata* infection is endemic in Cuba, *T. solium* is not.

In our study, all 37 samples had been preserved in 10% formalin from between less than 1 and almost 20 years. It is known that formalin-fixed samples can be difficult for molecular analysis due to cross-linking between nucleic acids and proteins (Okello et al., 2010) and also to DNA fragmentation due to the phosphodiester backbone disintegrating in the presence of formalin (Duval et al., 2010), both of which can block DNA amplification (Dietrich et al., 2013). However, in our study, the protocol we used for real-time PCR for *Taenia* (Praet et al., 2013) amplified all *Taenia*-positive samples. In a similar study in Korea, *Taenia* specimens preserved in 10% formalin that had been collected between 1929 and 1996, and therefore some of which had been in 10% formalin for over 80 years, were successfully analysed (Jeon et al.*,* 2011).

DNA extraction remains a critical factor in order to obtain genomic DNA of quality for molecular studies. Taleb-Hossenkhan et al. (2013) reported that the Qiagen DNeasy Blood & Tissue kit was suitable for DNA extraction from the parasitic tapeworm *Bertiella studeri* from formalin or ethanol-preserved tissues. However, the authors suggested that the DNA recovery yield could be lower in older specimens (Taleb-Hossenkhan et al., 2013).

The phylogenetic analysis of three of the *T. saginata* samples based on the cox1 gene indicated no significant genetic difference between our isolates and *T. saginata* from other countries.

## 5 Conclusions

Taeniosis is an important parasitic disease in Cuba, but it is reassuring that *T. solium* was not detected among the samples analysed here. The reason for the elevation in incidence, particularly in 2020, is worthy of further investigation.

Exploration of risk factors for infection with *T. saginata* in Cuba could be of value, along with closer examination of cattle for this parasite. Although it is mandatory that cattle are checked at slaughter for cysticerosis, reporting is scanty and the efficiency of the inspection in Cuba is unknown. It is also important to ensure that *T. solium* remains absent from Cuba, and we recommend that *Taenia* species is determined for all taeniosis cases reported in Cuba.

## Acknowledgements

We would like to thank our colleagues at the Institute for Tropical Medicine, Antwerp, Belgium for their assistance in obtaining the primers and other reagents for the PCR described herein.

## Declaration of Competing Interest

The authors declare that they have no known competing financial interests or personal relationships that could have appeared to influence the work reported in this paper.
